# α-Mangostin protects against high-glucose induced apoptosis of human umbilical vein endothelial cells

**DOI:** 10.1042/BSR20170779

**Published:** 2017-12-12

**Authors:** Yanli Luo, Minxiang Lei

**Affiliations:** Department of Endocrinology, Xiangya Hospital, Central South University, Changsha 410008, P.R. China

**Keywords:** α-mangostin, apoptosis, ceramide, high glucose, human umbilical vein endothelial cells

## Abstract

Diabetic vascular complications result from high-glucose induced vascular endothelial cell dysfunction. There is an emerging need for novel drugs with vascular endothelial cell protective effects for the treatment of diabetic vascular complications. The present study aimed to investigate the protective effect of α-mangostin against high-glucose induced apoptosis of cultured human umbilical vein endothelial cells (HUVECs). HUVECs were treated with glucose to induce apoptosis. The expression of the apoptosis-related proteins, Bcl-2, Bax, and cleaved caspase-3, were detected by Western blotting. Ceramide concentration and acid sphingomyelinase (ASM) activity were assayed by HPLC. The cell apoptosis rate was detected by flow cytometry after staining with annexin V/propidium iodide (PI). Compared with HUVECs cultured in 5 mM glucose, cells cultured in 30 mM glucose exhibited a higher apoptosis rate, up-regulation of cleaved caspase-3 and Bax (proapoptotic proteins), down-regulation of Bcl-2 (anti-apoptotic protein), increased ceramide concentration, and enhanced ASM activity (all *P*<0.05). α-Mangostin (15 µM) significantly attenuated the high-glucose induced increase in apoptosis rate (8.64 ± 2.16 compared with 19.6 ± 3.54%), up-regulation of cleaved caspase-3 and Bax, down-regulation of Bcl-2, elevation of ceramide level, and enhancement of ASM activity (all *P*<0.05). The effects of desipramine were similar to those of α-mangostin. The protective effect of α-mangostin on high-glucose induced apoptotic damage may be mediated by an inhibition of ASM and thus a decreased level of ceramide.

## Introduction

Diabetes mellitus, a metabolic disease characterized by hyperglycemia and insulin resistance, is a leading cause of deaths worldwide [1,2]. The global incidence of diabetes mellitus has increased year by year and is predicted to reach 4.4% in 2030, a substantial rise from the value of 2.8% reported in 2000 [3]. The increasing prevalence of diabetes mellitus has been attributed to several factors, including rapid industrialization/urbanization and associated changes in lifestyle [4,5].

Diabetic vascular complications, which can involve the blood vessels of the heart, brain, kidney, fundus, and other important organs, are the main cause of death and disability in diabetes [6]. The vascular diseases caused by diabetes are believed to result from impaired endothelial function. The mechanism of endothelial dysfunction in diabetes is complex, and is not fully understood, although it is thought to involve high-glucose induced oxidative stress [7], abnormal glucose metabolism [8], and/or protein kinase C pathway activation [9], resulting in an impairment of vascular structure and function. At present, the drugs commonly used in the management of diabetes are mainly aimed at reducing blood glucose levels and improving insulin resistance. Despite ongoing research into novel mechanisms that could be targetted to improve the dysfunction of vascular endothelial cells [10,11], there are still no drugs in clinical use that effectively treat diabetic microvascular disease [12]. The discovery of new drugs for the treatment of diabetic microvascular complications is of critical importance.

One of the manifestations of vascular endothelial cell injury is apoptosis [13], and there is now evidence that elevated cellular levels of ceramide and enhanced activity of sphingomyelinase may contribute to the induction of cell death. Since human umbilical vein endothelial cells (HUVECs) could be cultured and expanded *in vitro*, many studies on the functions of vascular endothelial cells have used HUVECs as a model [14–17]. Previous *in vitro* studies have shown that HUVECs cultured in high-glucose medium exhibited a significant increase in ceramide concentration that resulted in cell apoptosis [18]. Furthermore, increased plasma ceramide content and serum acid sphingomyelinase (ASM) activity have been observed in patients with type-2 diabetes mellitus [19,20]. Our previous study also found that ceramide levels in serum and aorta were elevated in a rat model of diabetes, and that blocking serine palmitoyltransferase with myriocin could reduce ceramide synthesis and inhibit atherosclerotic changes [21]. The regulation of apoptosis by ceramide may be due, at least in part, to actions in mitochondria [22]. In addition, ceramide is capable of up-regulating the level of the proapoptotic protein, Bax, and down-regulating the level of the anti-apoptotic protein, Bcl-2, thereby changing the ratio of Bax/Bcl-2 and promoting cell apoptosis [23,24].

α-Mangostin, a naturally occurring compound isolated from the bark and sap of the mangosteen tree (*Garcinia mangostana*), has been reported to have numerous biological effects, including anti-inflammatory, antioxidant, anti-allergic, antibacterial, and antitumor actions [25–28]. Several recent investigations have suggested that α-mangostin may also have beneficial actions in diabetes. For example, studies in rat models of diabetes have reported that α-mangostin was capable of reducing the early changes of diabetic retinopathy [29] and improving sexual dysfunction in male rats [30]. Moreover, rats with experimental type-2 diabetes that were fed α-mangostin showed improved glucose tolerance and had increased serum insulin levels [31]. Interestingly, the consumption of α-mangostin in mixed juice has been reported to reduce the body mass index of people with obesity [32]. Although the mechanisms underlying the potentially beneficial effects of α-mangostin in diabetes remain unknown, one study observed that α-mangostin could inhibit the activity of ASM [33], raising the possibility that this could play an important role.

Based on the above research, we hypothesized that α-mangostin protects against hyperglycemia-induced vascular endothelial cell dysfunction by inhibiting ASM activity and suppressing cell apoptosis. To test this hypothesis, the present study examined the concentration-dependent effects of α-mangostin on cellular ceramide level, ASM activity, and apoptosis of HUVECs cultured in high-glucose medium.

## Materials and methods

### Chemicals and reagents

HUVEC-12 were purchased from the Cell Culture Center of Xiangya Medical College, Central South University, China. Newborn calf serum and RPMI 1640 medium were obtained from Gibco (Carlsbad, CA, U.S.A.). d-glucose, l-glucose, α-mangostin, desipramine, Igepal CA-630, and cell lysates were sourced from Sigma (St. Louis, MO, U.S.A.). Naphthalene-2,3-phthalaldehyde, recombinant human acidic ceramide enzyme, and BODIPY-labeled C12-sphingomyelin were purchased from Molecular Probes (Eugene, OR, U.S.A.). Ceramide standard products were obtained from Matreya (Pleasant Gap, PA, U.S.A.). Protease inhibitors were sourced from Roche Diagnostics (Indianapolis, IN, U.S.A.). Antibodies against Bax, Bcl-2, β-actin, and glyceraldehyde 3-phosphate dehydrogenase (GAPDH) were purchased from Santa Cruz Biotechnology (Danvers, TX, U.S.A.). Antibodies against caspase-3 were obtained from Bioss Inc. (Woburn, MA, U.S.A.). The electrophoresis equipment was acquired from Bio–Rad (Hercules, CA, U.S.A.).

α-Mangostin was dissolved in DMSO to obtain stock solutions with concentrations of 5, 10, and 15 mM, which were stored at room temperature in the dark. To obtain α-mangostin-containing medium for use in the experiments, 1 µl of the appropriate stock solution was diluted in 2 ml RPMI 1640 medium containing 10% FBS. Desipramine was prepared as a 2 mM stock solution in ddH_2_O and stored at 4°C. For the experiments, medium that contained desipramine at a concentration of 2 µM was prepared by mixing 2 µl of the stock solution with 2 ml medium. The other reagents were used in accordance with the instructions provided by the manufacturers.

### HUVEC culture and treatment

HUVEC-12 were thawed, resuspended in RPMI 1640 medium supplemented with 10% FBS, and cultured in a culture flask. The cells were incubated at 37°C in a humidified environment containing 5% CO_2_. Approximately 5 × 10^5^ culture flask cells (1 × 10^5^ cells/ml) were seeded in a six-well plate or 25-cm^2^ culture flask and incubated with the indicated drugs for 24 h before further tests were performed. The cultured HUVECs were divided into seven groups: (i) 5 mM d-glucose group; (ii) 30 mM d-glucose group; (iii) 30 mM d-glucose + 5 µM α-mangostin group; (iv) 30 mM d-glucose + 10 µM α-mangostin group; (v) 30 mM d-glucose + 15 µM α-mangostin group; (vi) 30 mM d-glucose + 2 µM desipramine group (ASM inhibitor, used as a positive control); and (vii) 30 mM l-glucose group. Medium containing 30 mM d-glucose was chosen as the high-glucose culture environment based on a recent study [34]. α-Mangostin was added to the culture medium immediately after the addition of glucose; the α-mangostin concentrations used were based on a previous study [35]. The cells cultured under these conditions in culture flasks were used for the determination of cellular ceramide and the extraction of cellular proteins.

### Cell apoptosis assay

The adherent cells were digested with 0.25% trypsin (EDTA free) for approximately 2 min, washed twice with cold PBS, and then centrifuged for 5 min at 2000 rpm. The cells were resuspended at a density of 1 × 10^6^ cells/ml in 400 µl of 1× binding buffer, and 5 µl of annexin V-FITC (MB-CHEM, Shanghai, China) was added to the cell suspension. After gentle mixing, the cells were incubated for 15 min at 4°C in the dark. Then, 10 µl propidium iodide (PI) was added, and the cell suspension was incubated for 5 min at 4°C in the dark. The samples were analyzed by flow cytometry (FACSAria flow cytometer and accompanying software; BD Biosciences, San Jose, CA, U.S.A.) within 1 h.

### Determination of cellular ceramide levels

The determination of cellular ceramide was carried out according to the method described by He et al. [36]. After the cells had been cultured for 24 h in the appropriate medium, 25 µl of the supernatant was added to 150 µl of a mixture (1:2) of dichloromethane and methanol. After vortexing for 1 min, 100 µl of a 1-M solution of NaCl (dissolved in concentrated HCl) and 100 µl of dichloromethane were added. This mixture was vortexed for 1 min and then centrifuged for 1 min. The lower phase was transferred into a 1.5-ml centrifuge tube and centrifuged for 5 min. After evaporation at room temperature, 20 µl of 2% Igepal was added, and the mixture was then incubated at 80°C for 5 min, vortexed for 10 s, cooled to room temperature, and centrifuged at 13000 rpm for 20 s. A 2-µl volume of supernatant was transferred into a centrifuge tube, 2 µl ceramide substrate buffer was added, and the mixture was incubated for 1 h. Subsequently, 56 µl of naphthalene-2,3-dicarboxaldehyde (NDA) was added as previously described [37], and the mixture was vortexed at 50°C for 10 min and then centrifuged at 14000 rpm for 5 min after cooling. Using the buffer containing 0.2 M citrate/phosphate buffer pH 4.5, 0.3 M NaCl, and hiAC (1:10), a 35-µl volume of supernatant was transferred into the automatic sampling device and examined for the amount of ceramide automatically by an Acquity Ultra Performance Liquid Chromatography (UPLC) system equipped with an amide 1.7-µm reaction column (Waters, Milford, MA, U.S.A.). The volume examined was 5 µl at a time. The ceramide concentration was calculated as ceramide content/total protein concentration [36]. Column: ACQUITY BEH Shield RP18 1.7 µm 2.1 × 50 mm (186002853). Guard column: ACQUITY BEH Shield RP18 VanGuard Pre-column, 1.7 μm, 2.1 × 5 mm (186003977); 12 × 32 mm Amber glass screw neck vial with preslit PTFE_Silicone septa (Waters, # 600000669CV). One hundred fifty microliters of low volume glass inserts (Waters, # WAT072294). Mobile phase: 0.1% of concentrated NH_4_OH (Fisher, A669-500).

### Determination of ASM activity

The method used for the determination of ASM was the same as that has been reported previously [36]. A 3-µl volume of buffer (200 mM BODIPY-labeled C12-sphingomyelin, 200 mM sodium acetate buffer, pH 5.0, 200 µM ZnCl_2_, and 0.2% Igepal CA-630) was added to 3 µl of cell lysate in a centrifuge tube, and the mixture was incubated for more than 12 h in the dark. Then, 54 µl of ethanol was added, and the mixture was centrifuged for 5 min at 14000 rpm. A 35-µl volume of supernatant was transferred into the automatic sampling device and detected using an Acquity UPLC system with an amide 1.7-µm reaction column. The volume of detected sample was 5 µl at a time.

### Western blotting for Bcl-2 and Bax proteins

The cells were lysed in cOmplete™ CelLytic™ M buffer (Sigma, C2978) containing protease inhibitors. The samples were loaded with a ratio of sample buffer to sample volume of 5:1. Proteins were electrophoresed on 10% SDS/polyacrylamide gels and transferred into nitrocellulose membranes (Bio–Rad, Hercules, CA, U.S.A.). Membranes were blocked with 5% non-fat milk in PBS buffer and incubated with the following primary antibodies: anti-Bcl-2 (1:500), anti-Bax (1:300), anti-caspase-3 (1:200), anti-β-actin (1:800), and anti-GAPDH (1:8000), in accordance with the manufacturer’s instructions. Subsequently, the membranes were incubated with secondary antibodies. Immunoreactive bands were detected and analyzed using Gel Pro Analyzer 4.0 software (Media Cybernetics, Rockville, MD, U.S.A.).

### Reactive oxygen species level detection

Reactive oxygen species (ROS) levels were determined by ROS detection kit purchased from Beyotime (Shanghai, China), according to the manufacturer’s instructions.

### Cellular viability determination

Cell viability was determined by Trypan Blue staining.

### Statistical analysis

Data were analyzed using SPSS 18.0 statistical software (SPSS Inc., Chicago, IL, U.S.A.). Data are presented as the mean ± S.D. Statistical comparisons were made using one-way ANOVA, and the Dunnett’s *t* test was used for comparisons between two groups at the same time point. A *P*-value <0.05 was considered statistically significant.

## Results

### α-Mangostin reduces high-glucose induced apoptosis of HUVECs

**Figure 1:**
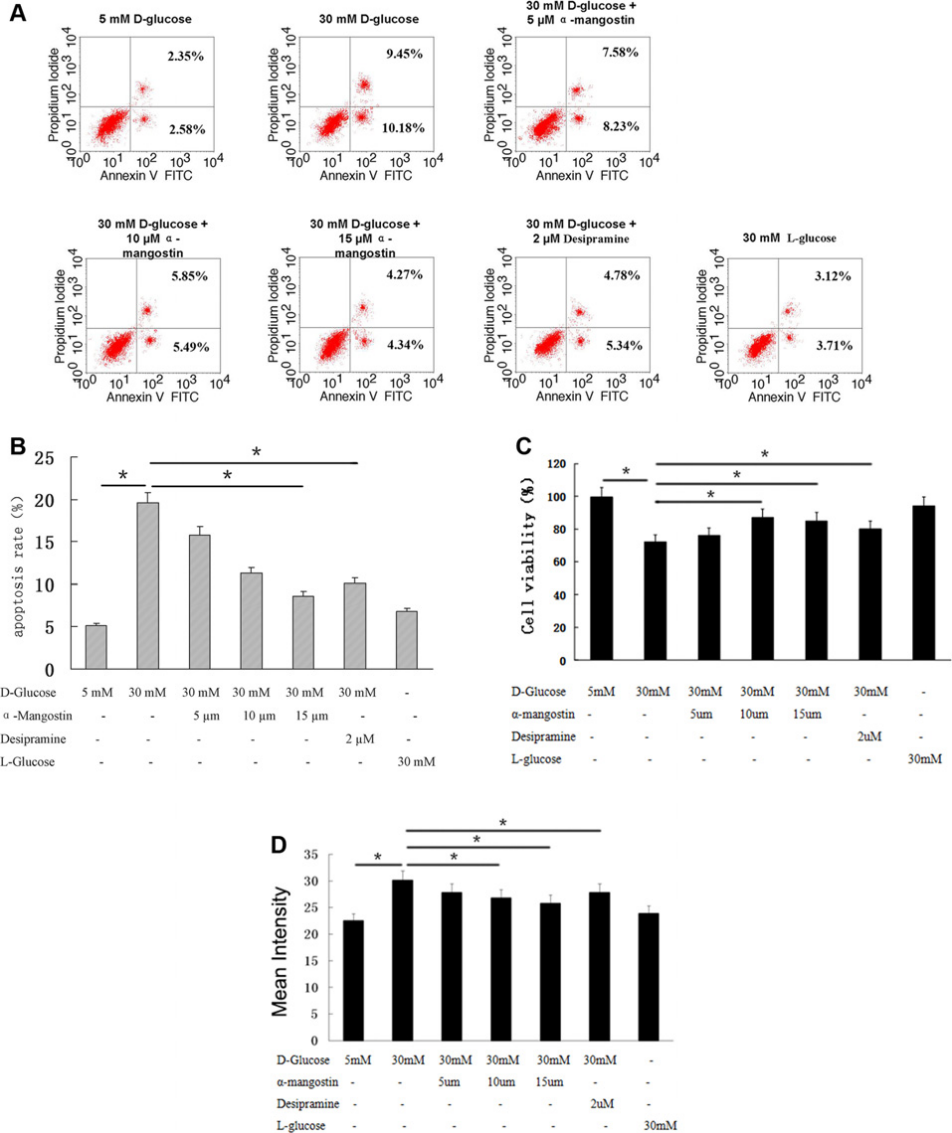
The effect of α-mangostin on high-glucose induced HUVEC apoptosis (**A**) Flow cytometry assessment of annexin V/PI staining to show apoptosis in HUVECs. (**B**) Statistical analysis of data shown in (A). (**C**) Cell viability was shown from (A). (**D**) ROS levels were shown from (A). (B–D) Data are representative of three independent experiments performed in triplicate. *P<0.05.

The apoptosis of HUVECs after culture for 24 h is shown in Figure 1. Compared with HUVECs cultured with 5 mM d-glucose, the apoptosis was significantly higher in cells cultured with 30 mM d-glucose (*P*<0.01), but not in cells cultured with 30 mM l-glucose. Here, l-glucose was chosen as a hypertonic control for 30 mM d-glucose, since l-glucose cannot be metabolized by these cells. This high-glucose induced apoptosis was reduced by α-mangostin in a concentration-dependent manner, with the apoptosis of cells cultured with 30 mM d-glucose + 15 µM α-mangostin significantly lower than that of cells cultured with 30 mM d-glucose alone (*P*<0.05) (Figure 1A,B). Desipramine is an ASM inhibitor [38] that can inhibit apoptosis induced by high glucose [39], possibly by activating Bcl-2 expression [40]. Here, as a positive control, 2 µM desipramine also caused a significant decrease in apoptosis in cells cultured with 30 mM d-glucose (*P*<0.05) (Figure 1A,B). However, the presence of α-mangostin in addition to desipramine did not decrease apoptosis levels further (results not shown). Similar changes in cell death were also observed (Figure 1C). Since the generation of ROS and oxidative stress are early events in the cell death process, we also measured the ROS levels of HUVECs, and found that α-mangostin, as well as desipramine, significantly decreased the ROS levels enhanced by d-glucose (Figure 1D), which was consistent with our observations on the apoptosis and cell death in HUVECs (Figure 1A–C).

### α-Mangostin counteracts high-glucose induced cleaved caspase-3 and Bax, and high-glucose induced decreases in Bcl-2 in HUVECs

**Figure 2:**
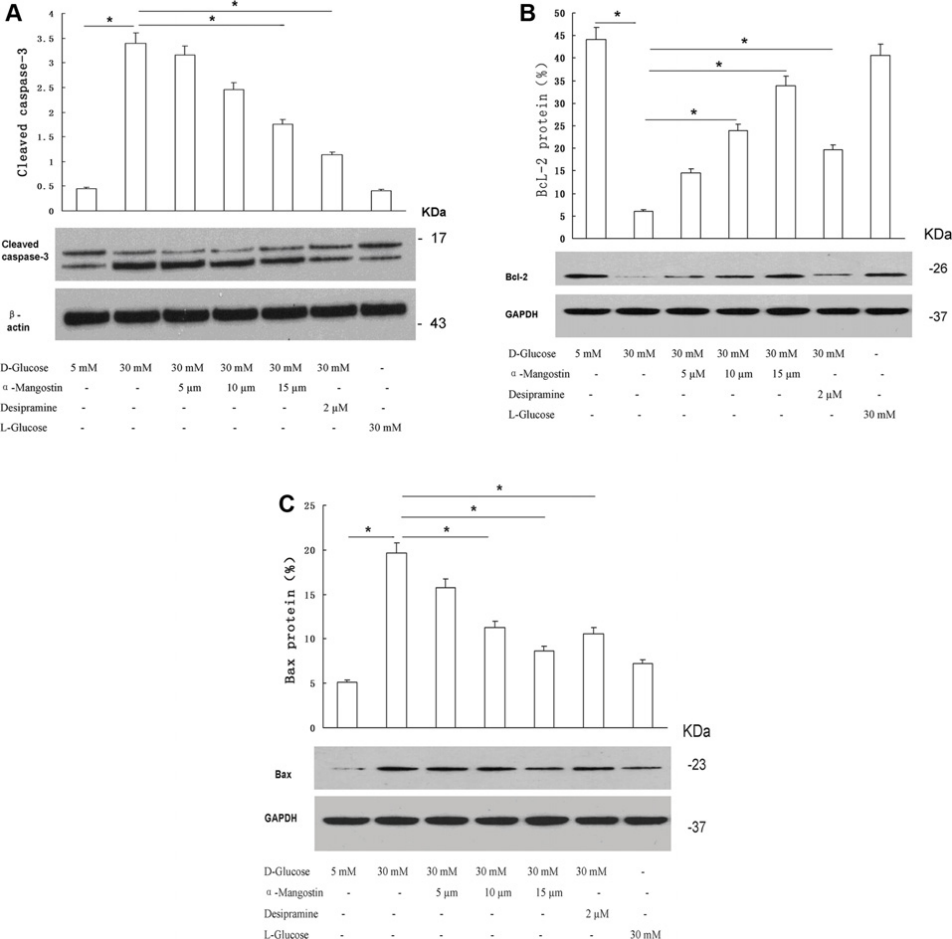
The effect of α-mangostin on high-glucose treated HUVEC expression of cleaved caspase-3, Bcl-2, and Bax Expression of cleaved caspase-3 (**A**), Bcl-2 (**B**), and Bax (**C**) was detected by Western blotting. The upper panels show representative immunoblots; the lower panels show the quantitative analysis. (A–C) Data are representative of three independent experiments performed in triplicate. *P<0.05.

The expression levels of cleaved caspase-3 in HUVECs cultured for 24 h are shown in Figure 2A. The expression of apoptosis marker, cleaved caspase-3, was significantly higher in HUVECs cultured with 30 mM d-glucose than in cells cultured with 5 mM d-glucose (*P*<0.01); in contrast, expression did not differ significantly between the 30 mM l-glucose and 5 mM d-glucose groups. For cells cultured with 30 mM d-glucose, the expression of cleaved caspase-3 was significantly decreased by the addition of 15 µM α-mangostin (*P*<0.05) or 2 µM desipramine (*P*<0.05) to the culture medium.

Comparisons of the expression levels of the anti-apoptotic protein, Bcl-2, between groups are presented in Figure 2B. Compared with cells cultured with 5 mM d-glucose, Bcl-2 expression was significantly reduced in cells cultured with 30 mM d-glucose (*P*<0.01), but not in cells cultured with 30 mM l-glucose. Bcl-2 expression in cells cultured with 30 mM d-glucose was significantly increased by the addition of 10 µM α-mangostin, 15 µM α-mangostin, or 2 µM desipramine to the culture medium (all *P*<0.05).

Figure 2C shows the expression levels of the proapoptotic protein, Bax, in HUVECs cultured for 24 h under the various experimental conditions. Bax expression was significantly elevated in the 30 mM d-glucose group compared with the 5 mM d-glucose group (*P*<0.01); in contrast, culture with 30 mM l-glucose did not increase Bax expression relative to that in 5 mM d-glucose. The up-regulation of Bax expression in 30 mM d-glucose was significantly attenuated by 10 µM α-mangostin, 15 µM α-mangostin, and 2 µM desipramine (all *P*<0.05). Taken together, the above results indicate that α-mangostin protects against high-glucose induced apoptosis in HUVECs.

### α-Mangostin counteracts high-glucose induced ceramide levels in HUVECs

**Figure 3:**
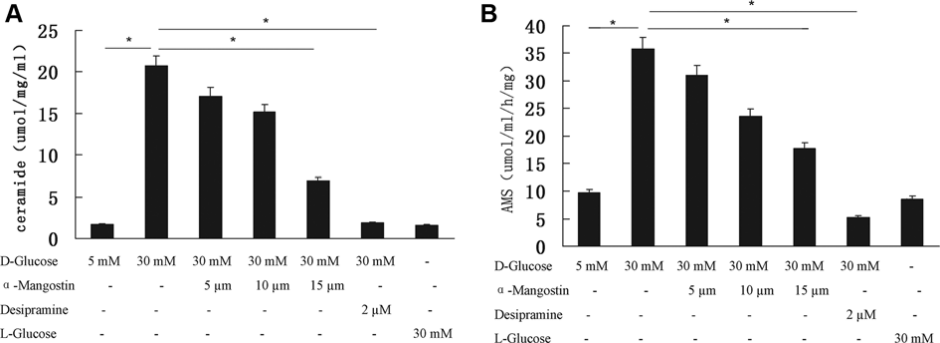
The effect of α-mangostin on high-glucose induced ceramide level and ASM activity in HUVECs (**A**) Ceramide levels. (**B**) Sphingomyelinase activity. (A,B) Data are representative of three independent experiments performed in triplicate. *P<0.05.

In order to investigate the mechanisms of action by α-mangostin, we assessed the possible apoptosis pathways that might be affected by α-mangostin. Ceramide generation is an essential step in apoptosis. Figure 3A summarizes the levels of ceramide in HUVECs after 24 h of culture for the various experimental groups. Increasing the concentration of d-glucose in the culture medium from 5 to 30 mM resulted in a significant elevation of ceramide level (*P*<0.01). The high-glucose induced increase in ceramide level was significantly attenuated by both 15 µM α-mangostin (*P*<0.05) and 2 µM desipramine (*P*<0.01). Ceramide levels were similar for the 5 mM d-glucose and 30 mM l-glucose groups.

### α-Mangostin counteracts high-glucose induced ASM activity in HUVECs

Sphingomyelinase is a mediator of apoptosis. The activity of the ASM in HUVECs cultured for 24 h under the various experimental conditions is shown in Figure 3B. ASM activity was significantly elevated when the d-glucose concentration was increased from 5 to 30 mM (*P*<0.01); in contrast, ASM activity was similar between the 5 mM d-glucose and 30 mM l-glucose groups. The increase in ASM activity induced by high glucose was significantly inhibited by 15 µM α-mangostin (*P*<0.05) and 2 µM desipramine (*P*<0.01).

## Discussion

The main finding of the present study was that 15 µM α-mangostin attenuated the increase in HUVEC apoptosis induced by high concentrations of d-glucose. Consistent with this effect, α-mangostin also attenuated high-glucose induced up-regulation of both cleaved caspase-3 and Bax (proapoptotic proteins) and high-glucose induced down-regulation of Bcl-2 (an anti-apoptotic protein). Importantly, α-mangostin also inhibited the increases in HUVEC ceramide levels and ASM activity that were induced by high glucose, further confirming its anti-apoptosis effects. To the best of our knowledge, this is the first report on the effect of α-mangostin to inhibit the apoptosis of HUVECs cultured under high glucose conditions via a mechanism that may involve inhibition of ASM activity and reduced ceramide levels. These novel findings establish a foundation for further clinical research into the development of new drugs to treat the vascular complications of diabetes mellitus.

Patients with diabetes show islet cell hypofunction, which leads to impaired glucose tolerance, an increase in blood glucose levels, and disturbances in lipid metabolism, macroangiopathy, and microangiopathy, resulting in vascular endothelial dysfunction and cell apoptosis. Hyperglycemia has been demonstrated to cause irreversible damage to the vascular endothelium, and induce the apoptosis in HUVECs [41,42]; the results of the present study are consistent with these previous reports. Therefore, interventions that inhibit endothelial cell apoptosis would be expected to reduce the risk of diabetic vascular complications. α-Mangostin is a naturally occurring xanthonoid that has been shown to have beneficial effects in animal models of diabetes [29–31], as well as in people with obesity [32]. Lee et al. [43] reported that α-mangostin could inhibit contrast agent induced apoptosis of renal tubular epithelial cells, while Fang et al. [44] reported that α-mangostin could attenuate oxidative stress induced retinal cell death. Consistent with both these investigations, our study observed that α-mangostin could inhibit high-glucose induced apoptosis of HUVECs in a concentration-dependent manner.

Ceramide is an intracellular second messenger that is involved in various cellular functions such as cell proliferation, differentiation, and growth inhibition [45]. However, high-glucose levels can induce an increase in cellular ceramide levels, and the abnormal accumulation of ceramide can induce cell apoptosis, one of the main factors contributing to diabetic angiopathies [46]. In agreement with previous investigations, this study found that exposure of HUVECs to high glucose resulted in an increased cellular ceramide level. Furthermore, we observed that α-mangostin was able to inhibit the increase in ceramide concentration in HUVECs cultured in high glucose, raising the possibility that the anti-apoptotic effects of α-mangostin are, at least in part, due to a reduction in ceramide levels.

Desipramine is an ASM inhibitor [38] that can inhibit apoptosis induced by high glucose [39]. Moreover, the anti-apoptotic action of desipramine is thought to be due to an inhibition of ASM and a reduction in ceramide [47]. Since α-mangostin has been reported to inhibit ASM activity [33], it is possible that the anti-apoptotic actions of α-mangostin and desipramine may share similar mechanisms. It was notable that, in our study, the effects of 2 µM desipramine on apoptosis rate and expressions of apoptosis-related proteins were very similar to those of 15 µM α-mangostin. Furthermore, we directly observed that α-mangostin decreased ceramide levels and ASM activity in HUVECs exposed to high glucose, consistent with the results reported by Okudaira et al. [33].

To date, very few studies have explored the inhibition of ASM by naturally occurring products. To the best of our knowledge, this is the first study to show that α-mangostin can inhibit ASM, reduce ceramide levels, and decrease apoptosis in HUVECs exposed to high glucose. ASM mainly exists in the lysosomal membrane, where it can hydrolyze sphingomyelin to generate ceramide and phosphorylcholine – this is one of the main sources of rapid ceramide production [47]. Our findings suggest that α-mangostin might serve as a potential inhibitor of ASM, thereby reducing ceramide concentration and exerting an anti-apoptotic effect. However, further research is required to establish the precise mechanisms involved.

Current study reveals the protective effect and mechanisms of α-mangostin on high-glucose exposed HUVECs at the cellular and molecular level, shedding light on the potential of α-mangostin to treat diabetic vascular complications. Further studies are required to investigate whether α-mangostin also exerts a beneficial effect on vascular endothelial injury in animal models of diabetes.

## Abbreviations

ASM: acid sphingomyelinase
Bax: Bcl-2 associated X Protein
BCL-2: B-cell lymphona 2
GAPDH: glyceraldehyde 3-phosphate dehydrogenase
hiAC: human acid ceramidase
HUVEC: human umbilical vein endothelial cell
ROS: reactive oxygen species
UPLC: ultra performance liquid chromatography
